# Fabrication of silver helix microstructures in a large area by a two-photon absorption DLW method

**DOI:** 10.1038/s41598-021-95457-x

**Published:** 2021-08-04

**Authors:** Naoto Tsutsumi, Yusaku Takai, Kenji Kinashi, Wataru Sakai

**Affiliations:** 1 Faculty of Materials Science and Engineering, Kyoto Institute of Technology, Matsugasaki, Sakyo, Kyoto, 606-8585 Japan; 2 Master’s Program of Innovative Materials, Graduate School of Science and Technology, Kyoto Institute of Technology, Matsugasaki, Sakyo, Kyoto, 606-8585 Japan

**Keywords:** Chemistry, Engineering, Materials science, Nanoscience and technology, Optics and photonics, Physics

## Abstract

Large-area helix microstructures intended for metamaterials were fabricated using a negative photoresist, SU-8 using a two photon absorption direct laser writing (TPA-DLW). Two types of helix structures were fabricated. One type is those with no neighboring distance. In this case, compact helix structures with radius of 2.5 and 1.0 μm were fabricated. Another type is those with enough neighboring distance. The helix structures with shorter neighboring distance below 6.0 μm were collapsed, whereas those with longer neighboring distance more than 6.5 μm, free-standing helix structures could successfully be built. To stabilize the fabricated free-standing helix microstructures with a 1 μm radius, circular foundations with a radius of 1.3 μm and elevation angle of 10, 12, or 14° were built in advance. The foundation is useful to avoid collapsing the helix microstructures. Due to the useful foundation, over 18,000 helical structures were fabricated in a large area. The fabricated helical structures were coated with silver using an electroless plating method to produce 3D metallic helix structures. Silver coating was measured using a EDX measurement. The obtained helical structures have the potential for metamaterials to control the handedness of a circularly polarized infrared beam.

## Introduction

Metamaterials consisting of artificially engineered periodical metal structures can control electromagnetic waves, including visible light, and they provide fascinating functions of negative refraction^1–4^, invisibility cloaking^5–7^, and perfect superlenses^8^ that do not occur in conventional natural materials. Pendry et al. proposed a split-ring resonator (SRR), i.e., a ring with a slit, for metamaterials^9^. Micrometer- to submicrometer-scale engineering is required to fabricate metamaterials working in the wavelength region from visible to infrared^10^. Direct laser writing (DLW) with two-photon polymerization (TPP) and two-photon absorption (TPA) is a powerful tool to fabricate complexed three-dimensional (3D) microstructures using commercially available positive^11^ and negative photoresists^12^. Photo reduction of metal ions is also used^13,14^.

Spiral, helical and omega microstructures are fabricated using TPA-DLW for broadband circular polarizers^15–19^. In previous studies on spiral and helical 3D metamaterials on the micrometer scale, compact, dense, intricate, and bichiral microstructures were used to avoid collapsing the fabricated microstructures^16,17^. A twisted omega microstructure was supported by two points on the substrate^19^. In past studies, the fabrication of large-scale micrometer metamaterials on the order of mm^2^ has not been reported. Then we targeted to fabricate the large-scale micrometer metamaterials on the order of mm^2^ using a TPA-DLW method. Recently, a new type of DLW method using a phase mask of a double helix on a spatial light modulator (SLM) was proposed, in which the single-exposure femtosecond photoreduction of silver ions was used to fabricate the silver helix metamaterial^20^.

In this report, we present the fabrication of more than 18,000 free-standing 3D helical structures on the micrometer scale over a total area on the mm^2^ scale by TPA-DLW. The key point for large area fabrication is to stabilize the fabricated free-standing helix microstructures supported by one point on the substrate. For this purpose, we introduced the foundation underneath the fabricated helix microstructure in advance, which usefully avoids collapsing the fabricated helix microstructures. Furthermore, the neighboring distance between the helix microstructures is also an important parameter to avoid collapsing the structures. Electroless silver plating^17,19,21^ was employed in the present study. Another plating method, such as electrode gold plating^15^ or chemical vapor deposition^22^, can also be employed.

## Experimental section

### Materials

SU-8 50 (Kayaku Advanced Materials, formerly Microchem, USA) was used for microstructure fabrication. SU-8 50 is a negative photoresist consisting of epoxy monomer, photo acid emitter, and solvent.

### Sample preparation

The negative photoresist SU-8 50 was spin-coated on a glass substrate at 3000 rpm for 60 s to obtain a sample film with a thickness of 30–60 μm. The obtained spun-coated film was soft baked at 65 °C for 10 min followed by 95 °C for 50 min.

### Fabrication of helix microstructures

**Figure 1:**
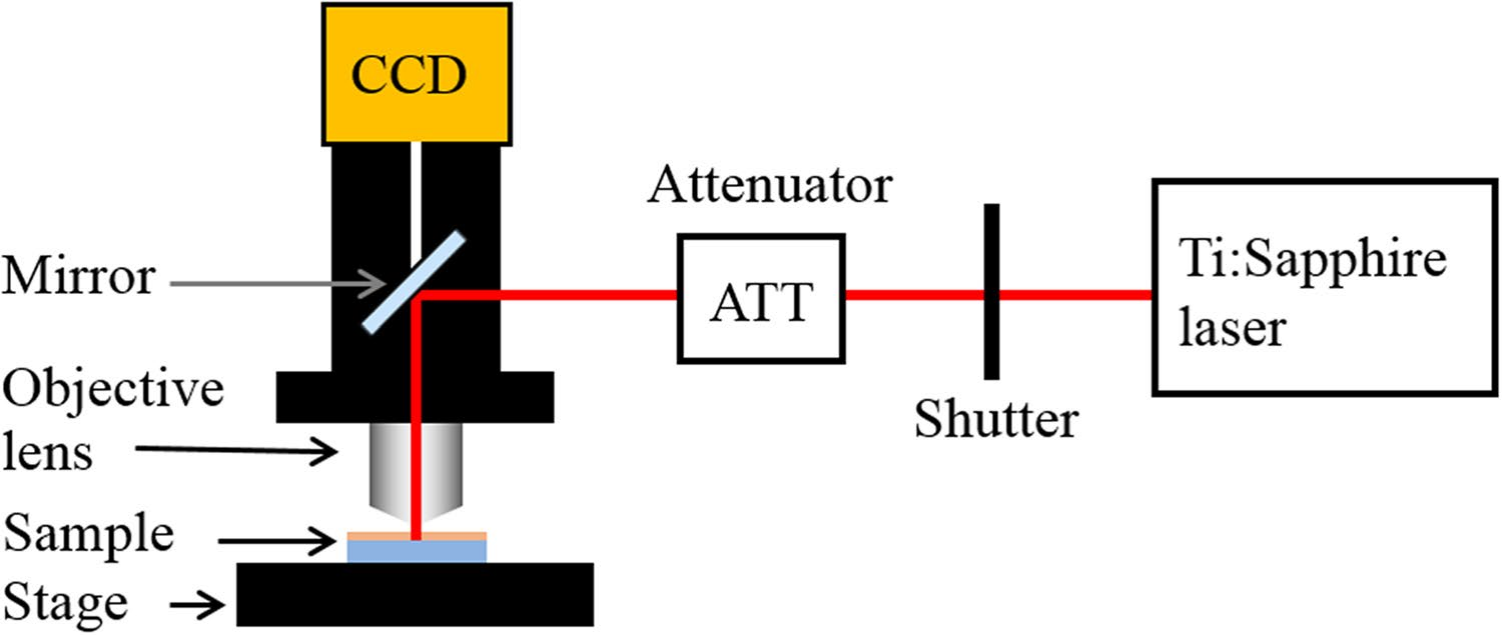
Schematic apparatus of the DLW system.

**Figure 2:**
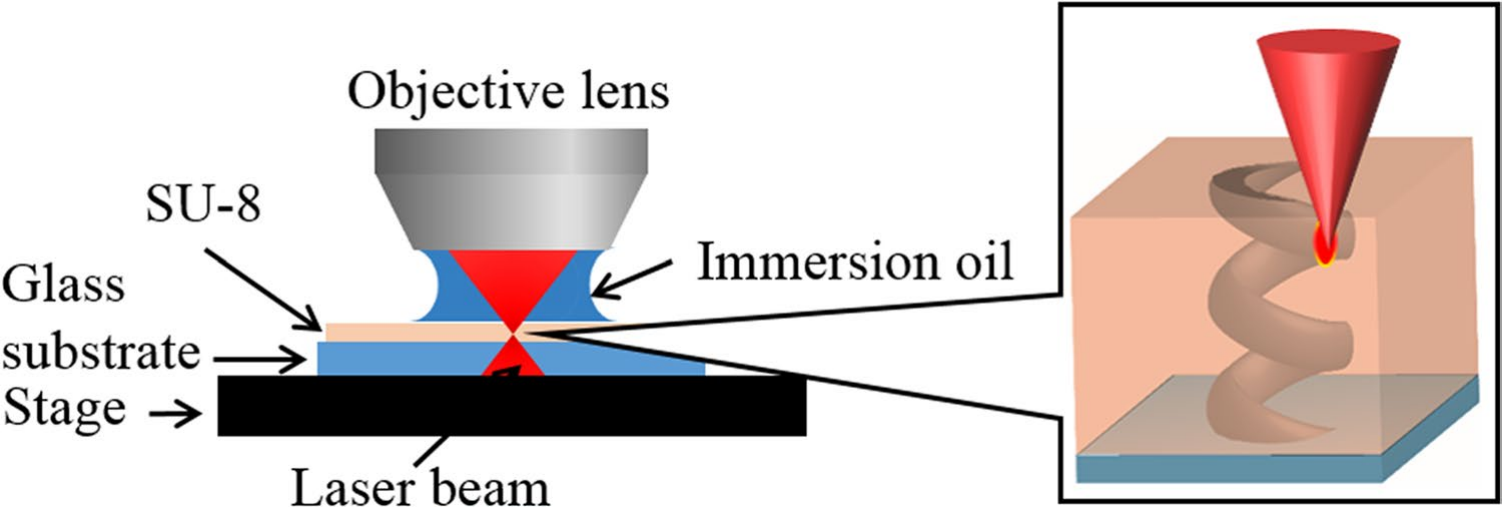
Detailed schematic of the objective lens, immersion oil, SU-8, and glass substrate including the focusing point. Illustration on the right shows the helix microstructure fabricated by DLW in the SU-8 negative photoresist.

A Ti:sapphire laser (Spectra Physics, Mai Tai wavelength: 800 nm, pulse width: 100 fs, repetition rate: 80 MHz) was used as the DLW laser source. The femtosecond laser beam was introduced to an Olympus microscope BX61WI equipped with an oil-immersion objective lens (Olympus UPlan FLN, ×100, NA = 1.30). The three-dimensional helix photonic metamaterial was fabricated inside an SU-8 film on Newport VP-25XA-XYZ stages controlled by ALPS 3861. The travel range of each stage is 25 mm with 100 nm resolution. The intensity of the laser power is attenuated by an attenuator (ATT), and the scanning (writing) speed of the laser is 20 μm s^−1^. A schematic of the DLW apparatus shown in Fig. 1. Figure 2 shows a detailed schematic of the objective lens, immersion oil, SU-8, and glass substrate, including the focusing point. The illustration on the right shows the helix microstructure fabricated by DLW in the SU-8 negative photoresist.

Three-dimensional structures whose pattern was already built by a computer software were fabricated using the DLW system. After laser fabrication, a postexposure bake (PEB) was performed at 65 °C for 10 min and at 95 °C for 50 min to complete the cross-linking reaction, followed by development for 10 min in an SU-8 Developer (Kayaku Advanced Materials, USA) to obtain the given structures. After development, the obtained structures were rinsed with 2-propanol (Nacalai Tesque, Japan) and dried under ambient conditions.

### Hydrophilization of fabricated helix structures

Before electroless silver plating, the surface of the fabricated helix structures was hydrophilized. Two types of hydrophilic method were employed. One is hydrophilization of epoxy moiety of SU-8 with a 2-aminoethanol. SU-8 sample was immersed in 2-aminoethanol at 50 °C followed by rinsing in water and drying in nitrogen flow of 110 ml min^−1^ during overnight. The other is that using a UV ozone cleaner (Filgen, UV253H(R), Japan). The oxygen flow rate was 6.0 L min^−1^, and the treatment time was 60 min. The hydrophilicity of the sample surface was improved as follows: UV light-induced reactive oxygen attacked the sample surface to form hydrophilic species of OH, CHO, or COOH. After hydrophilic treatment, the contact angle of the surface of SU-8 samples was measured to determine the proper method of the hydrophilic treatment.

### Electroless silver plating

The substrate with the fabricated helical structures was dipped into a tin(II) chloride solution (0.25 mg of SnCl_2_, 1 mL of hydrochloric acid, and 14 mL of deionized water) and rinsed in deionized water. Then, the substrate with the fabricated helical structures was immersed in the mixture solution of a silver nitride solution (0.15 M AgNO_3_) with a small droplet of 0.2 M ammonia solution and 0.19 M glucose solution (in 30 vol% methanol and 70 vol% deionized water) to complete the silver plating. After plating, the substrate with the fabricated helical structures was rinsed in deionized water and 2-propanol to terminate the plating.

### Microscope observation

The fabricated structures were observed using a scanning electron microscope (SEM) (Hitachi S-3000, Japan), and a field-emission SEM (FE-SEM) (JEOL JSM-7600F, Japan). Platinum was sputtered on the sample to give conductivity using one ion sputter (Hitachi E-1010, Japan) and another ion sputter (JEOL JFC-1600, Japan).

### Characterization

Elemental analysis was performed using energy dispersion X-ray (EDX) (Hitachi S3000N, Japan and Oxford INCA Energy).

To check the hydrophilicity of the treated surface of the obtained SU-8 structures, the contact angle of the surface was measured using deionized water. In that case, a separately prepared thin SU-8 film was used to evaluate the contact angle. The preparation conditions were the same as those for the structure fabrication except for the use of a UV lamp (AS ONE wavelength: 365 nm) instead of a femtosecond laser.

## Results and discussion

### Evaluation of hydrophilization

First we evaluated the condition of the electroless plating on the surface of SU-8. Because of the hydrophobicity of SU-8 photoresist, we faced the difficulty of electroless plating of SU-8 photoresist. To dissolve the difficulty, the hydrophilic treatment of the surface of SU-8 was employed. Two types of hydrophilic method were employed as discussed in experimental section. The hydrophilicity of the SU-8 surface was evaluated by the contact angle of water. The hydrophilic treatment by immersing in 2-aminoethanol led the reduction of contact angle from 90° for an untreated SU-8 surface to 61.5–72.0° for a treated SU-8 surface for 20 min. Further immersing time did not reduce the contact angle. Next hydrophilic treatment of the SU-8 surface was performed in a UV ozone cleaner. The contact angle of water on an untreated SU-8 surface is 90°, whereas that on a treated SU-8 surface is 9.5–11.7°. UV zone treatment drastically alters the surface hydrophilicity. Then, we employed the hydrophilic treatment in UV ozone cleaner before the electroless silver plating was performed.

### Electroless plating

**Figure 3:**
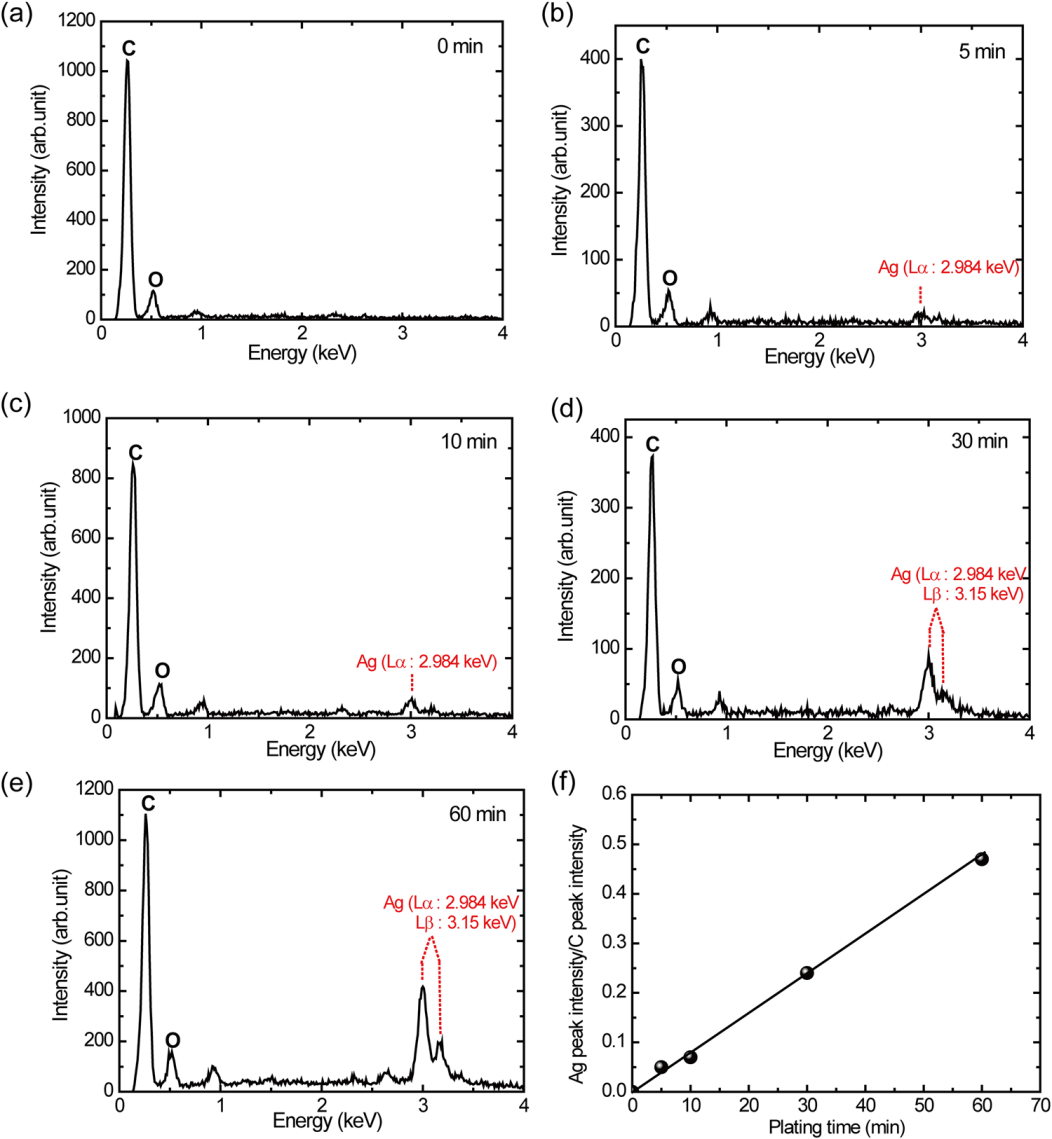
EDX spectrum of the SU-8 surface after electroless silver plating. (**a**) Before plating at t = 0 min. (**b**) After 5 min of plating. (**c**) After 10 min of plating. (**d**) After 30 min of plating. (**e**) After 60 min of plating. (**f**) Plot of the peak ratio between Ag and C as a function of plating time.

In the process of electroless silver plating, the silver content was varied by changing the immersion time from 5 to 60 min. To check the Ag content on the SU-8 surface, the EDX spectrum was measured after electroless silver plating for various periods of time. Figure 3 shows the EDX spectrum for various immersion times (plating time) from 5 to 60 min. In Fig. 3f, the peak intensity ratio between the peak intensity for silver (Ag) and that for carbon (C) is plotted as a function of plating time. The ratio almost linearly increases with plating time. This result implies that the surface of SU-8 was successfully plated by silver atoms.

### Helix structures fabricated with no neighboring distance

**Figure 4:**
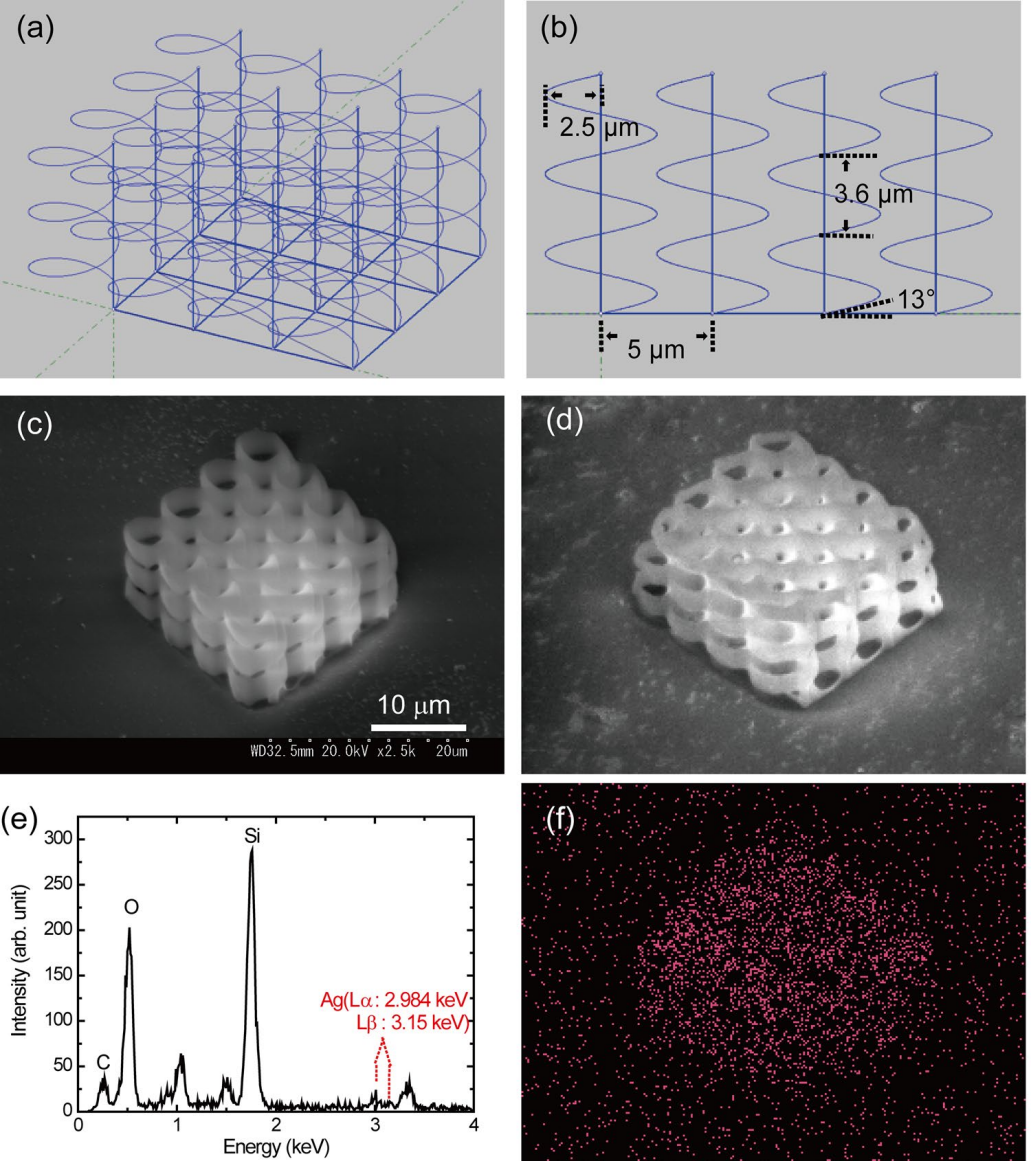
(**a**) Programmed pattern of a helical structure with a radius of 2.5 μm and an elevation angle of 13°. (**b**) Cross section of the programmed pattern shown in (**a**). (**c**) SEM image of fabricated helical structures using the programmed pattern in (**a**) before electroless silver plating. (**d**) SEM image of the fabricated helical structures after electroless silver plating. (**e**) EDX spectrum of the fabricated structure with silver plating. (**f**) EDX image of Ag in the fabricated helical structure with silver plating.

A helical structure with a radius of 2.5 μm, elevation angle of 13°, and number of helix pitches of 3 was fabricated using a laser power of 7 mW. The interval between helix is 5 μm with no neighboring distance. Figure 4 shows the programmed pattern, SEM image of fabricated helical structures before and after electroless silver plating, EDX spectrum and image of silver (Ag). Each helical structure is compact and close to a neighboring helix, which prevents collapse.

**Figure 5:**
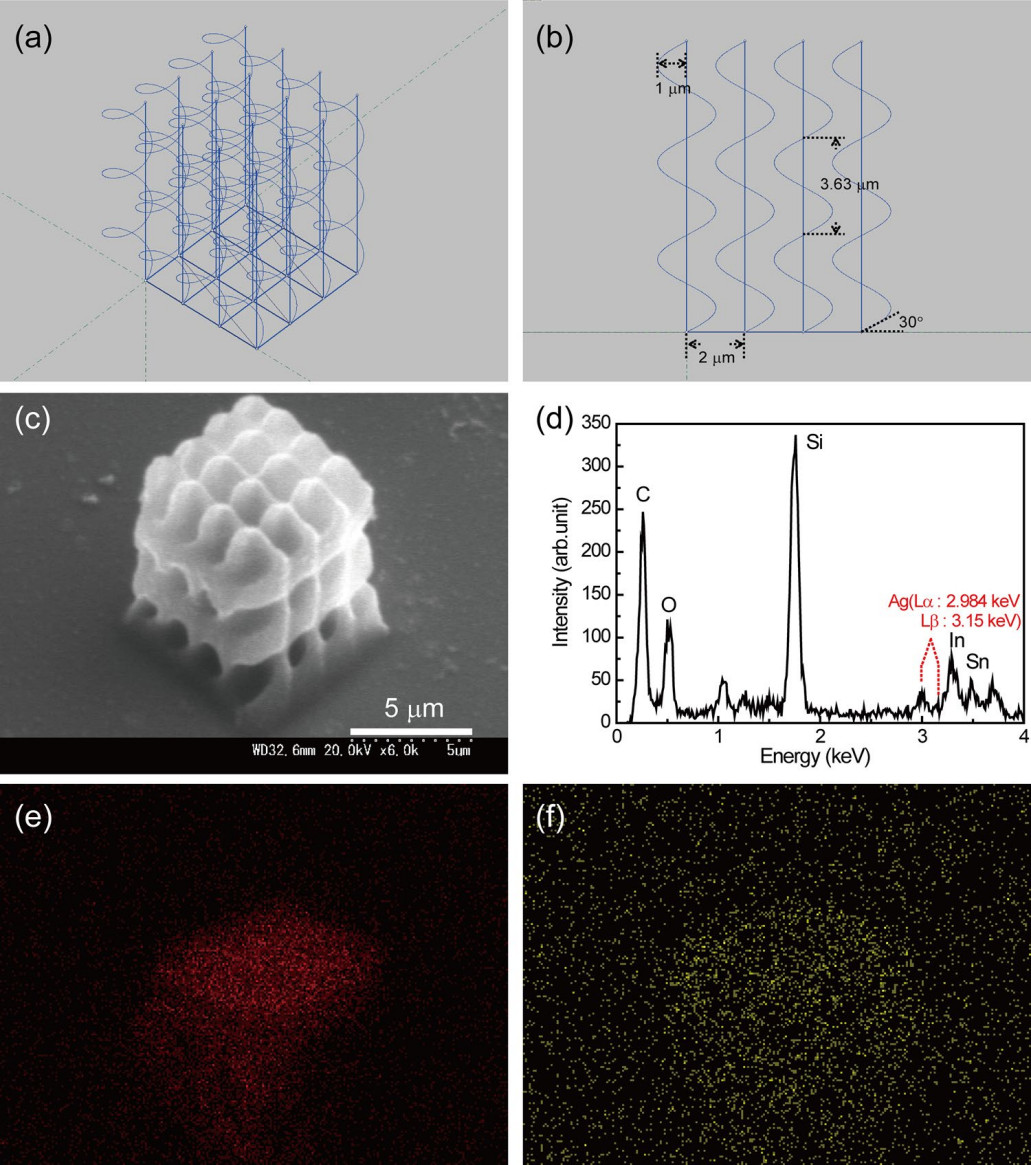
(**a**) Programmed pattern of a helical structure with a radius of 1.0 μm and an elevation angle of 30°. (**b**) Cross section of the programmed pattern shown in (**a**). (**c**) SEM image of the fabricated helical structures. (**d**) EDX spectrum of the fabricated structure with silver plating. (**e**) EDX image of C in the fabricated helical structure with silver plating. (**f**) EDX image of Ag in the fabricated helical structure with silver plating.

Next, helical structures with a radius of 1 μm, elevation angle of 30°, and number of helix pitches of 3 was fabricated using a laser power of 6.5 mW. The interval between helix is 2 μm with no neighboring distance. Figure 5 shows the programed pattern, SEM image of fabricated helical structures after electroless silver plating, EDX spectrum and EDX images of carbon (C) and silver (Ag). Each helical structure is close to a neighboring helix, which prevents collapse.

### Helix structures fabricated with enough neighboring distance

**Figure 6:**
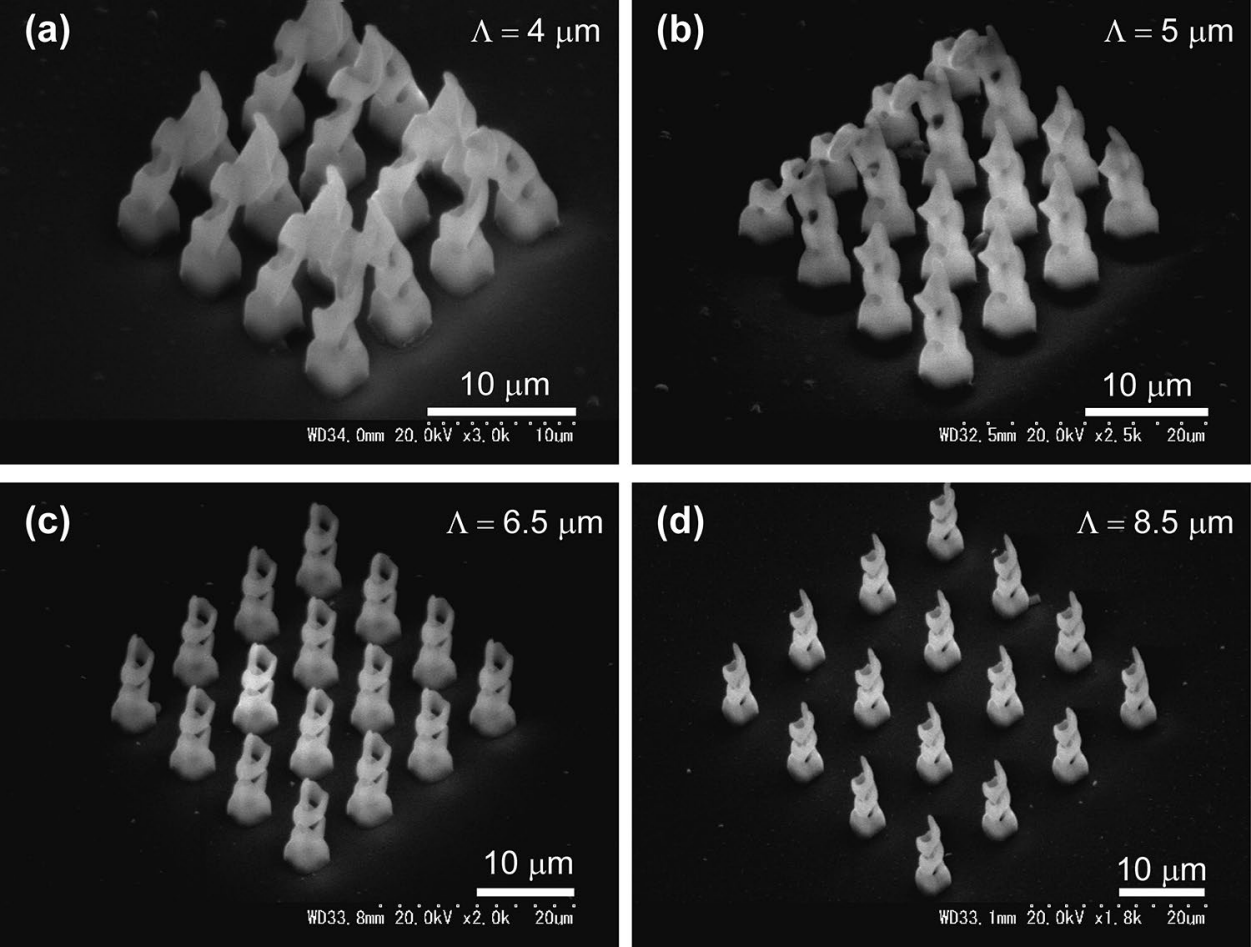
SEM images of the fabricated helix structures with various neighboring distance of 4 μm in (**a**), 5 μm in (**b**), 6.5 μm in (**c**), and 8.5 μm in (**d**).

The helical structures shown in Figs. 4 and 5 are close to each other. Next, helical structures with a radius of 1 μm and with enough neighboring distance are fabricated. In that case, the foundation is required to prevent collapse of the fabricated helical structure. The first foundation layer consisted of three circular with an each radius of 1, 1.25, and 1.5 μm was built at the distance of 0.9 μm above the substrate and the second foundation layer of three circular with an each radius of 1, 1.25, and 1.5 μm at the distance of 0.9 μm above the first layer. On the double layered foundation, 16 helix structures with an each radius of 1 μm, elevation angle of 30°, and number of pitch of 3 was built with the neighboring distance, *Λ*, between helix structures from 4.0 to 9.5 μm on the double layered foundations. Figure 6 shows the SEM image of the fabricated helix structures. For the sample with the neighboring distance, *Λ*, from 4.0 to 6.0 μm, the fabricated helix structures were collapsed in the rinsing process even though the existence of the foundation. This may be caused by the capillary action of 2-propanol between helix structures in the rinsing process. Shorter distance between helix structures led stronger capillary pressure to collapse the neighboring helix structures.

**Figure 7:**
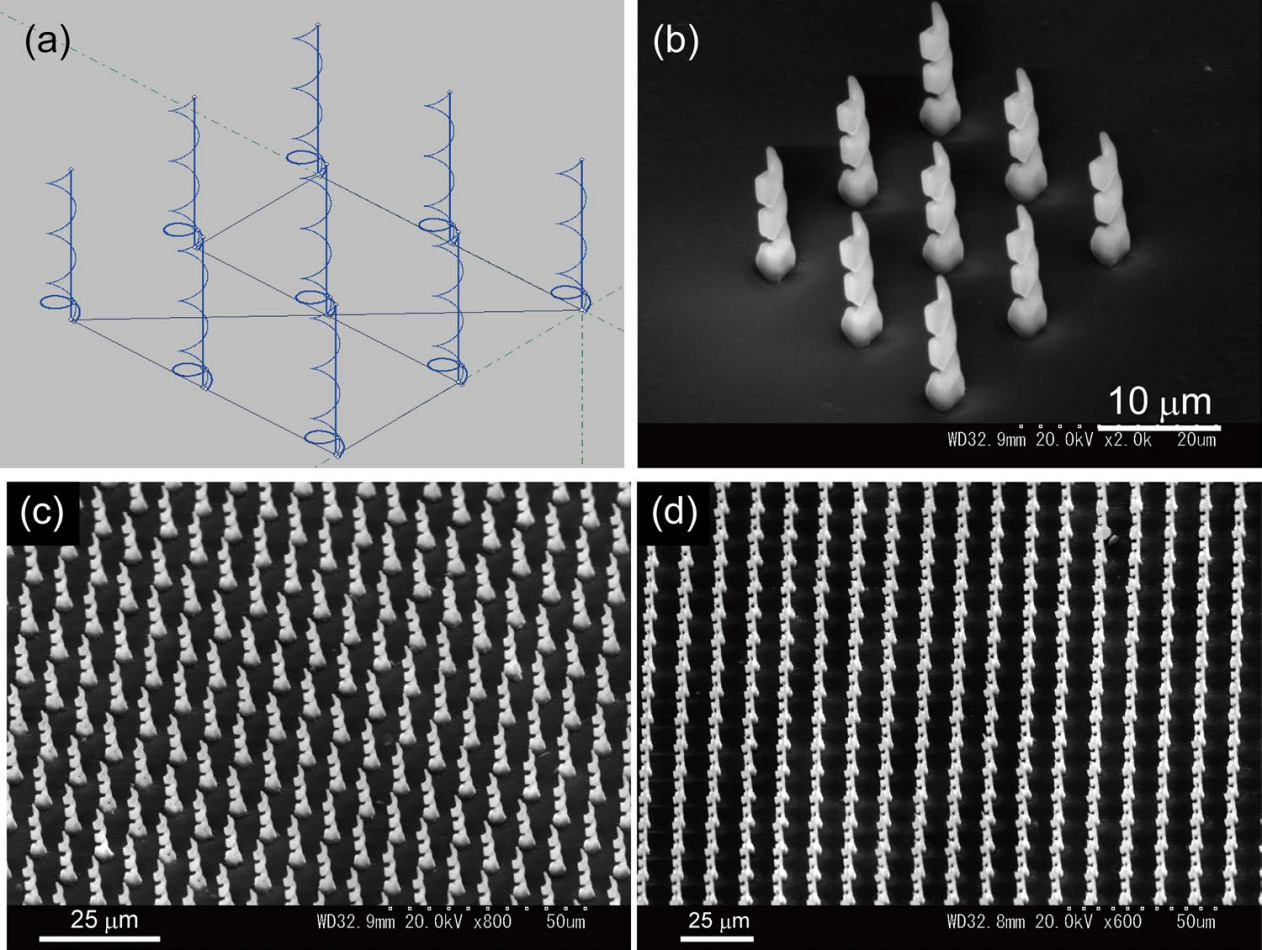
(**a**) Programmed pattern of 9 helical structures. (**b**) SEM image of 9 practically fabricated helical structures using the programmed pattern in (**a**). (**c**,**d**) SEM image of the helical structures fabricated in a wide area by repeating DLW of the 1900 helical structures (38 line × 50 line).

Furthermore, it took longer period of time to fabricate the double layered foundation, and these time lead to the time consuming (time loss) for fabricating the structures with larger area. Double layered foundation was changed to simple circular foundation with an elevation angle. Thus, a circular foundation with a radius of 1.3 μm and elevation angle of 10, 12 or 14° was built. The laser power was 13 or 14 mW. Then, a helical structure with a radius of 1 μm, elevation angle of 40°, and number of pitches of 3 was built on the foundation. Figure 7 shows the programmed pattern of the helical structure and SEM image of the practically fabricated stand-alone helical structures. The programmed diameter of the helix is 2 μm, and the practical diameter of the fabricated helical structure is measured to be 3.3 μm from the SEM image. The enlarged diameter is due to the finite width of the laser beam, diffraction limit, and diffusion of the photochemically produced photoacid by the TPA process. A total of 1900 free-standing helical structures with a radius of 1 μm and neighboring distance of 10 μm (38 lines × 50 lines) were fabricated. SEM images of part of the 1900 helical structures are shown in Fig. 7c,d.

### End-fire helical antenna

A total of 18,260 helical structures with a radius of 1 μm and neighboring distance of 10 μm (22 lines × 830 lines) were fabricated. The total area is 2.50 mm^2^. For the end-fire helical antenna, the wavelength (*λ*) sensitive to circularly polarized infrared light is related to the helix pitch (Λ) with the relation of $\Lambda \approx \lambda /4$. A helix pitch is calculated by an elevation angle of helix (*θ*): $\theta ={{\tan}}^{-1}\left(\Lambda /2\pi R\right)$, where *R* is the radius of the helix. Thus, the sensitive wavelength can be

$$\lambda =4{\tan}\theta \times 2\pi R$$

The present helical structure has a radius of 1 μm (diameter 2 μm) and an elevation angle of 40°. In this case, the sensitive wavelength can be calculated to be 21.1 μm (470 cm^−1^). Because of the IR absorption of the glass substrate in the wavelength region of 20 μm, the selective circular polarization of the fabricated helical structures cannot be investigated. Alternatively, the use of a reflective aluminum-coated substrate and/or IR transparent substrate such as KRS5 will promise the achievement of the measurement. Furthermore, the reduction in diameter or the reduction in elevation angle will promise the optical performance of the end-fire helical antenna working in the wavelength region of 5–10 μm.

## Conclusions

In the present report, the TPA-DLW method is used to fabricate large-area helical structures using a negative photoresist. To stabilize the fabricated free-standing helix microstructures with a 1 μm radius, a circular foundation with a radius of 1.3 μm and elevation angle of 10, 12, or 14° was constructed in advance. The foundation is useful to avoid collapsing the helical structures. Due to the useful foundation, over 18,000 helical structures are fabricated in a large area. The fabricated helical structures would be sensitive to the wavelength of the infrared region of 21 μm.
